# Assessment of oral health literacy with dental hygiene among rural communities in India

**DOI:** 10.6026/973206300210845

**Published:** 2025-04-30

**Authors:** Chanchal Gangwar, Anshul Gangwar, Pallavi Patel

**Affiliations:** 1 Department of Public Health Dentistry, Institute of Dental Sciences, Bareilly, Uttar Pradesh, India; 2 Department of Paediatric and Preventive Dentistry, Institute of Dental Sciences, Bareilly, Uttar Pradesh, India

**Keywords:** Oral health literacy, dental hygiene, rural communities, oral health behavior, public health dentistry

## Abstract

Oral health literacy (OHL) and its effect on hygiene practices among 300 adults in rural Bareilly, India is assessed. The average OHL score was 12.6 ± 3.5 out of 20, indicating moderate literacy. A strong positive correlation (r = 0.61, p < 0.001) was found between OHL and good oral hygiene practices. Only 34% brushed twice daily and 19% used mouthwash regularly. Higher OHL was significantly linked to regular dental check-ups (p = 0.002), highlighting the need for better oral health education in rural areas in India.

## Background:

The condition of one's mouth constitutes an integral aspect of overall health together with life satisfaction. A large percentage of the population across rural regions alongside other specific areas faces preventable oral diseases because of their inadequate oral hygiene behavior and lack of dental care opportunities [1]. According to research OHL stands as an emerging oral health behavior eterminant since it refers to a person's capability of grasping essential information needed to select suitable healthcare choices [2]. People with inadequate oral health literacy tend to face adverse oral health impacts such as dental caries occurrences alongside periodontal disease development and tooth loss [3]. People who have little information about oral health tend to avoid preventive oral care steps which include brushing teeth, flossing and visiting the dentist [4]. The health problems in rural areas become worse due to financial obstacles and insufficient awareness and constrained opportunities for oral health education [5]. Evidence based research indicates that boosting OHL strength leads patients to practice better oral hygiene habits thus lowering the number of dental diseases [6]. Many developing countries including India have insufficient data about the oral health literacy status of rural residents despite increasing awareness of its significance. Knowledge about the degree of OHL together with its consequences on hygiene behaviors remains crucial for designing successful community-based oral health interventions. The ability to understand dental pamphlets along with brushing directions represents only a basic aspect of oral health literacy because it includes larger concepts about disease prevention coupled with dietary understanding and dental check-up requirements [7]. People with sufficient OHL skills demonstrate better ability to comprehend oral health messages while making proper health-related choices and maintaining productive communication with dental specialists [8].

Toxic literacy generates poor health information comprehension thereby triggering health mistakes and substandard preventive service use [9]. The oral disease loads in rural areas grow worse because education levels and financial stability are lower and healthcare systems lack proper infrastructure [10]. Oral health in these areas demonstrates a reactive system because residents typically use traditional medicine unless experiencing severe dental problems before seeing professionals [11]. Unguided AIDS programming throughout these areas causes both decreased health knowledge and improper oral care methods resulting in advanced disease diagnoses. Community health interventions need to be developed based on thorough knowledge of how oral health literacy correlates to regular oral hygiene practices including brushing teeth and visiting a dentist for checkups [12]. Most health programs today focus on treating existing conditions rather than preventing them as they implement few empowerment strategies that depend on literacy education. Including OHL assessments in standard community health questionnaires enables healthcare teams to discover comprehension deficits which direct cultural programming for educational content [13]. The programs will maintain greater reach and sustainability whenever local healthcare workers along with school educators participate in the outreach for sharing oral health knowledge [14]. Long-term oral health improvement requires immediate research which evaluates both the nature of OHL knowledge and its effects on rural population behavior. Therefore, it is of interest to evaluate the oral health literacy standing of rural community adults with emphasis on literacy impacting their daily dental hygiene activities. This will also offer essential information which policy creators together with medical educators can utilize to create specific plans for oral health promotion in underserved communities.

## Materials and Methods:

A research investigation adopted a community-based cross-sectional approach to measure oral health literacy levels together with their impact on dental hygiene practices of rural area adult residents. Researchers studied this population throughout three months among adults within the 18 to 65 years age range.

## Study population and sampling technique:

Residents at three rural communities throughout Bareilly formed the study population of this study. The research used stratified random sampling because it helped achieve sufficient participation from various age brackets along with different social economic groups. Three hundred participants were selected after satisfying the inclusion requirements which included being a permanent local from selected areas who understood the local dialect and agreed to participate with informed consent. People who were mentally unable to comprehend or were currently receiving dental care were barred from inclusion in the study.

## Data collection tool:

The researchers pre-tested the questionnaire on thirty subjects from the target population to ensure reliability and validity. Three sections appeared in the questionnaire design.

[1] The research collected data about subjects' demographic characteristics which included their age, gender together with education level, occupation type and personal income.

[2] The assessment of Oral Health Literacy comprised questions from the Oral Health Literacy-Adult Questionnaire (OHL-AQ) portable version to measure reading comprehension with dental knowledge and numeracy skill.

[3] The dental hygiene section included questions on brushing routines as well as mouthwash usage and flossing habits and dental appointment schedules for the participants.

## Ethical considerations:

An approval for ethical research procedures came from the Ethical Review Board, Institute of Dental Sciences and Bareilly. Before starting data collection every participant provided written consent to participate. Participants received protection of both their confidential information and their identities from beginning to end of the research period.

## Statistical analysis:

The team processed their gathered information within Microsoft Excel and executed their analysis through SPSS version 25.0. Urns such as mean and standard deviation and frequencies with percentages came from the descriptive statistical approach. The investigation of OHL scores and hygiene practices relationship used Pearson's correlation test. The Chi-square statistical tests determined whether different categories exist among variables. The research used a p-value below 0.05 to establish statistical significance.

## Results:

Three hundred participants were included in the final study analysis. The research participants averaged 37.4 years old with a standard deviation of 11.2 years and female participants made up 58 percent (n = 174). Male participants amounted to 42 percent (n = 126). Most participants' finished primary education while secondary education earning second place and higher education ranking third. The demographic breakdown and oral health literacy results of participants appear in Table 1. Out of a maximum possible score of 20 the participants received an average of 12.6 ± 3.5 points which divided the participants into three literacy categories: 48% moderate literacy, 32% low literacy and only 20% high literacy. As shown in Table 2, individuals with higher OHL scores reported significantly better oral hygiene behaviors. Among participants with high OHL, 76.6% reported brushing twice daily compared to 28.1% in the low literacy group (p < 0.001). The use of mouthwash and dental floss was also more prevalent among those with moderate to high OHL. Regular dental visits were reported by 58.3% of participants with high literacy, whereas only 14.6% of low-literacy individuals reported the same (p = 0.002). These findings indicate a clear, statistically significant association between oral health literacy and positive dental hygiene practices (Table 2). Participants with higher literacy were more likely to engage in preventive behaviors, demonstrating the importance of OHL in promoting better oral health outcomes.

## Discussion:

This research study evaluated oral health literacy levels in rural adults and their connection to oral hygiene practices. Research data showed that a major segment of the population demonstrated both moderate or insufficient oral health literacy and these groups practiced poor oral hygiene routines. The current findings agreed with various previous studies that demonstrate the leading influence of OHL on oral health behaviors and outcomes [1, 2- 3]. The rural community showed moderate levels of oral health information understanding based on the observed mean score of 12.6 ± 3.5. The local health department needs provide community-level oral health education/interventions, such as washing mouth with clean water at least twice a day, teeth brushing using indigenous methods such as toothbrush sticks or modern methods such as toothpastes, and avoiding gum pricking to promote oral health [4]. Various studies within low-resource settings describe these same findings because patients experience limited dental healthcare access which leads to reduced literacy rates [5].

Participants who showed higher literacy levels performed all recommended oral hygiene activities which included regular tooth brushing combined with mouthwash use and tooth flossing along with consistent dental visits. The research proves that people with acceptable OHL levels practice proactive oral healthcare behaviors as well as seek routine preventive dental services more often [6, 7-8]. Educational attainment shows significant effects on oral health literacy as one of the main findings from this study. Individuals who received education beyond high school earned better scores on OHL evaluations and followed oral hygiene instructions without issue. The research supports the established theoretical connection between educational background and health literacy which appears in published literature [9]. Researchers are encouraged to incorporate oral health conceptual knowledge into their theoretical frameworks, especially as it relates to beliefs and self-efficacy [10]. Women participants' demonstrated marginally superior oral hygiene practices in this study because they exhibited higher health-seeking behavior according to other reported studies [11]. The weak relationship between poor OHL and decreased preventive dental habits supports the requirement for specific interventions to improve knowledge. Oral Health education must be imparted to preschool and primary school teachers as a part of National Oral Health care Program on a regular basis and further studies must be done to assess their awareness levels and make the necessary changes in further education modules [12]. Especially teachers have a positive attitude and were willing to train and help [13]. Programs based in communities should consider implementing ways to improve OHL by using visual materials and mobile dental services and training local healthcare providers to address literacy gaps in rural populations [14]. Strategies enhancing delivery must address barriers around training access, knowledge attrition, and variability in baseline skills through sustainable system-wide policies applied nationally [15]. Despite delivering useful findings this research study contains specific constraints. Social science research design limits the ability to prove cause-and-effect relationships between variables. The data about hygiene practices which people report might include bias because individuals tend to want to present themselves in a positive light. While the study has limitations, they make a necessary contribution that will support upcoming longitudinal investigations about culturally adapted educational intervention development.

## Conclusion:

Oral health literacy fundamentally influences hygiene practices of rural areas' residents. Specific and accessible educational programs for OHL development will potentially lead to better oral health results together with reduced dental disease impacts in underserved communities.

## Figures and Tables

**Table 1:** Demographic profile and oral health literacy scores of study participants (n = 300)

**Variable**	**Category**	**Frequency (%)**
Age (Mean ± SD)	-	37.4 ± 11.2
Gender	Male	126 (42.0%)
	Female	174 (58.0%)
Education Level	Primary	117 (39.0%)
	Secondary	99 (33.0%)
	Higher	84 (28.0%)
Oral Health Literacy Score	Low (≤9)	96 (32.0%)
	Moderate (10-15)	144 (48.0%)
	High (>15)	60 (20.0%)

**Table 2:** Association between oral health literacy and dental hygiene practices

**Oral Hygiene Practice**	**Low OHL (n = 96)**	**Moderate OHL (n = 144)**	**High OHL (n = 60)**	**p-value**
Brushing Twice Daily (%)	28.1	55.6	76.6	< 0.001
Use of Mouthwash (%)	11.4	30.6	53.3	< 0.001
Use of Dental Floss (%)	6.3	21.5	41.6	0.004
Regular Dental Visit (%)	14.6	37.5	58.3	0.002
